# Effect of Progesterone Supplementation on Natural Frozen-Thawed Embryo Transfer Cycles: A Randomized Controlled Trial

**Published:** 2013-03-06

**Authors:** Maryam Eftekhar, Mozhgan Rahsepar, Elham Rahmani

**Affiliations:** 1 Yazd Research and Clinical Center for Infertility, Shahid Sadoughi University of Medical Sciences, Yazd, Iran; 2 Department of Obstetrics and Gynecology, Faculty of Medicine, Bushehr University of Medical Sciences, Bushehr, Iran

**Keywords:** Progesterone, Pregnancy Rate, Embryo Transfer, Natural Cycle

## Abstract

**Background:**

The transfer of cryopreserved embryos can be timed with ovulation in a natural cycle or after artificially preparing the endometrium with exogenous hormones. Progesterone is essential for the secretory transformation of the endometrium that permits implantation as well as maintenance of early pregnancy. The purpose of this study is to assess the effect of luteal phase supplementation on pregnancy rates in natural frozen-thawed cycles.

**Materials and Methods:**

The study was designed as a prospective randomized clinical trial of 102 women who underwent embryo transfers in natural cycles. The women in the interventional group (n=51) received intra muscular (IM) progesterone 50 mg twice a day starting from 36 hours after hCG administration. The control group (n=51) did not receive any progesterone support.

**Results:**

There were no significant differences in demographic characteristics between the groups and no statistically significant differences were observed between study and control groups in clinical pregnancy rate (33.3% vs. 27.5%, p=0.66). There were no differences in implantation rate or spontaneous abortion rate.

**Conclusion:**

Our results suggest that luteal phase support does not affect clinical pregnancy rates in natural frozen-thawed embryo transfer cycles (Registration Number: IRCT201108044339N6).

## Introduction

Cryopreserved-thawed embryo transfer began in 1983 and became a popular, vital component of assisted reproduction technology (1). The transfer of a frozen embryo enhances the cumulative pregnancy rate, decreases cost, Is easy to perform and can be fulfilled successfully in a relatively shorter time span in comparison with repeated fresh cycles (2-5). Furthermore, endometrial receptivity can be compromised by controlled ovarian hyperstimulation (COH) protocols (6) and secretory endometrial transformation (7). Endometrial development in frozen-thawed cycles can be controlled more than during COH cycles (8).

Various protocols (gonadotropin/GnRH agonists, clomiphene citrate, or exogenous estrogen and progesterone) have been discussed in literature reviews with regards to the endometrium preparation for frozen-thawed embryo transfer (3, 9). The most prevalent protocol for frozen-thawed embryo transfer is the natural cycle or endometrial preparation with exogenous estrogen and progesterone, with or without the addition of a GnRH agonist (10-12).

Because the natural cycle protocol does not require exogenous hormones‚ it is favored by many patients (13). It has been observed that temporal characteristics of the endometrium such as the formation of pinopodes (markers of endometrial receptivity) are out-of-phase according to measurements in normal females who have been placed on exogenous steroids (14). Thus, the transfer of frozen-thawed embryos in natural cycles is a favored option for women with normal ovulatory menstrual cycles (15).

There is an idea that the endogenous production of progesterone is enough to support implantation in a natural cycle. However, an inadequate progesterone level at the time of implantation or during early pregnancy may happen naturally due to luteal phase deficiency (LPD), which can result in infertility or abortion (16).

The reported frequency of LPD ranges from 3.7% to 20% among infertile patients (17, 18). The frequency has been demonstrated to be approximately 8.1% in natural cycles in normoovulatory patients with primary or secondary infertility (19). Thus, women who undergo frozen- thawed embryo transfers may have sub optimal endometrium during their natural cycles. There is limited information about the effect of luteal phase supplementation on pregnancy rate in natural frozen-thawed embryo transfer cycles. Therefore, we have designed a prospective randomized study to verify if pregnancy rates could be enhanced with progesterone supplementation during the luteal phase and early pregnancy following a frozen-thawed embryo transfer in a natural cycle.

## Materials and Methods

### Study design

The study was designed as a prospective randomized clinical trial. A total of 102 women each underwent an embryo transfer in a natural cycle in Yazd Research and Clinical Center for Infertility affiliated by Shahid Sadoughi University of Medical Sciences, from March 2011 to March 2012. This study was approved by the Ethics Committee of Yazd Research and Clinical Center for Infertility. Prior to starting the study‚ an informed consent was signed by each couple. The inclusion criteria were: cryop reserved embryos after conventional *in vitro* fertilization (IVF) or intracytoplasmic sperm injection (ICSI)‚ maternal age of 20-40 years (on the day of embryo freezing)‚ regular menstrual cycle of 25-35 days, and body mass index of 20-27 kg/m^2^. Exclusion criteria were: the use of testicular sperm for ICSI (ejaculated sperm only)‚ basal follicle stimulating hormone (FSH) levels ≥12 IU/l, stage III-IV endometriosis, and polycystic ovarian syndrome (PCOS).

### Randomization

Patients were randomized to either group in a ratio of 1:1 by means of computer-generated random numbers on the day of participation. Group selection and randomization were performed by a nurse not involved in the study, by using opaque sealed envelopes. Both the patients and the clinicians were aware of the allocated arm.

### Treatment protocol

Of the initial 109 women invited to participate, 102 were included in the study. All women had previously undergone IVF or ICSI with embryo cryopreservation. They were randomly allocated to either the progesterone (n=51) or the no-progesterone (n=51) groups. In the progesterone group, we excluded four women. One patient had an endometrial polyp and three patients had thin endometria. Similarly, three patients were excluded from the no-progesterone group because of endometrial polyps (2) and one patient who did not return to the study. Thus, in this study, 102 women each underwent an embryo transfer in a natural cycle. The final analysis was performed on 51 patients in each group. On the second or third days of the menstrual cycle, all patients underwent transvaginal ultrasounds and serum hormone analysis for FSH. Then, a vaginal ultrasonographic examination was performed on cycle days 10 and repeated as necessary. Final oocyte maturation was achieved by intramuscular (IM) administration of 10000 IU of hCG (Pregnyl, Daropakhsh, Iran) when an endometrial thickness of 8 mm or more and a follicle of 18 mm were present on the ultrasound. On the day of the hCG administration, we measured serum estradiol‚ progesterone and LH levels.

The progesterone group received 100mg/day of progesterone (Aburaihan Pharmaceutical Co., Tehran, Iran) IM, that began 36 hours after the hCG administration and continued until ten weeks of gestation if pregnancy occurred. Control patients received no progesterone. In both groups, cryopreserved embryo transfer was performed with a Cook catheter (Cook Ireland Ltd.) five days after hCG administration. Serum β-hCG level was measured 14 days after the transfer.

### Embryo freezing-thawing

Morphology of fresh cleavage-stage embryos was evaluated according to the number of blastomeres and degree of fragmentation. Embryo selection for transfer or freezing was performed in the morning of the transfer day. Embryos were considered suitable for freezing if they had <30% fragmentation. Cryopreservation of all embryos was undertaken with vitrification by the cryotop method on day 3 of pre implantation development in both groups. After two-step loading with equilibration solution that contained ethylene glycol and dimethyl sulfoxide and a vitrification solution that contained ethylene glycol, dimethyl sulfoxide and sucrose, a narrow glass capillary was used to load the embryos onto the cryotop. After loading, the majority of the solution was removed to leave only a thin layer that covered the embryos, after which the sample was quickly immersed into liquid nitrogen. Subsequently, the plastic cap was pulled over the film part of the cryotop and the sample stored in liquid nitrogen. At warming, the protective cap was removed from the cryotop while it was still submerged in liquid nitrogen and the cryotop was immersed directly into a 37˚C medium that contained sucrose. Next, the embryos were sequentially incubated in diluent solution before further *in vitro* culture for transfer. Each embryo was carefully evaluated immediately after thawing for the number of surviving blastomeres, followed by a second evaluation the next morning. Embryos were accepted for transfer if they retained ≥50% of intact blastomeres after thawing.

### Outcome measures

The main outcome measures concerned clinical pregnancy and implantation rates. Chemical pregnancy was defined as serum β-hCG>50 IU/L at 14 days after the embryo transfer. Clinical pregnancy was defined as the presence of a gestational sac with heart beat identified by ultrasound 4-5 weeks after the embryo transfer. Implantation rate was defined as the ratio of gestational sacs to the number of embryos transferred. Clinical abortion rate was determined as clinically recognized pregnancy losses before 20 weeks of gestation.

### Statistical analysis

The SPSS 19 package program was used to perform all statistical analyses. The normality of distribution of variables was tested by the Kolmogorov- Smirnov test. Independent sample t test was used for continuous variables which were normally distributed and Mann-Whitney U test for data not normally distributed. Chi-square or Fisher exact tests were used for qualitative variables as appropriate. A p value <0.05 was considered statistically significant. The data are presented as the mean ± standard deviation unless otherwise indicated.

## Results

There were no significant differences noted in the fertilization rate between study and control groups (55.4% vs. 64.3%; p=0.16). Of the 102 patients included in this study, 51 received progesterone and the other 51 did not. Table 1 describes the basic characteristics of the patients in the two groups. The demographic parameters were similar in both groups in terms of age, basal FSH levels‚ body mass index (BMI)‚ the number of previous cycles‚ etiology of infertility, and infertility duration. Table 2 compares the previous fresh cycle characteristics in the two groups.

The mean number of oocytes retrieved‚ mean number of mature oocytes and the number of embryos obtained and vitrified did not differ between the groups. There were no significant differences noted in the fertilization rate (55.4% vs. 64.3%; p=0.16). In addition, the previous stimulation protocols and fertilization procedures were similar in the two groups. Only ejaculated sperms had used for conventional IVF or intracytoplasmic sperm injection and percent of sperms with progressive motility and sperms with normal morphology‚ also sperm count were not different in those groups. There was no significant difference observed between the groups regarding the reasons for embryo freezing. Table 3 compares the cycle characteristics of the two groups. Endometrial thickness and estradiol‚ progesterone and LH levels on the day of hCG administration were similar between groups.

**Fig 1:**
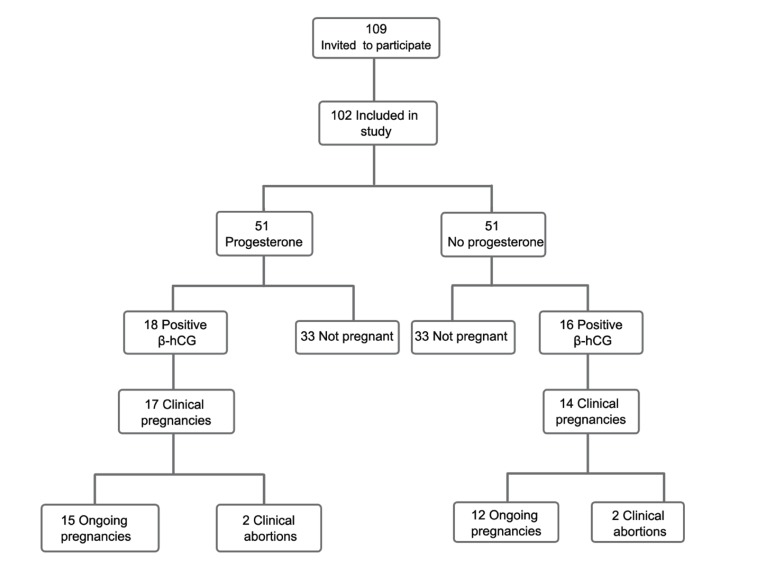
Flowchart of study patients

The cycle length until the day of hCG administration‚ number of embryos transferred, and the number of good-quality embryos did not differ in the two groups. Table 4 presents a comparison of the pregnancy outcomes of the study groups. Again, no statistically significant differences were observed in the clinical pregnancy rate between the groups (33.3% vs.27.5%, p=0.66). Although there was a trend toward an increased clinical pregnancy rate with luteal supplementation‚ the difference was not significant. There were no differences between the implantation rates (16.6% vs. 15.3%‚ p=0.93) or clinical abortion rates (11.8% vs.14.3%‚ p=0.83). The flowchart of the study is shown in figure 1.

**Table 1:** Characteristics of patients


Outcome variable	Progesterone N=51	No progesterone N=51	P value

**Age (Years)**	29.0 ± 3.8	28.7 ± 4.6	0.71
**BMI (kg/m^2^)**	23.8 ± 2.8	24.3 ± 2.4	0.35
**Duration of infertility (Years)**	6.0 ± 3.8	6.7 ± 4.5	0.71
**Basal FSH (IU/L)**	5.8 ± 1.9	6.0 ± 2.0	0.90
**Previous ART attempts n (%)**	14 (27.5)	17 (33.3)	0.51
**Etiology of infertility n (%)**			0.62
**Male factor **	35 (68.6)	32 (62.7)	
**Tubal factor **	7 (13.8)	6 (11.8)	
**Unexplained**	9 (17.6)	13 (25.5)	


**Table 2:** Patients’ previous fresh cycle characteristics


Outcome variable	Progesterone N=51	No progesterone N=51	P value

**Type of previous stimulation n (%) **			0.84
**Agonist protocol**	29 (56.9)	31(60.8)	
**Antagonist protocol**	22 (43.1)	20 (39.2)	
**Fertilization procedure n (%)**			
**IVF**	11 (21.6)	19 (37.3)	
**ICSI**	40 (78.4)	32 (62.7)	
**No. of oocytes retrieved**	10.0 ± 4.3	9.6 ± 3.4	0.16
**No. of mature oocytes**	8.3 ± 3.4	7.6 ± 2.8	0.22
**No. of embryos obtained**	6.2 ± 1.6	5.7 ± 2.2	0.19
**No. of embryos vitrified**	4.3 ± 1.0	4.0 ± 0.6	0.07
**Fertilization rate (%)**	55.4	64.3	0.16
**Sperm parameters**			
**Count (mill/ml)**	12.6 ± 7.7	11.9 ± 7.0	0.62
**Progressive motility (%)**	15.0 ± 5.8	14.5 ± 6.9	0.72
**Normal morphology (%)**	15.3 ± 9.8	14.2 ± 7.3	0.51
**Cause of embryo freezing n (%) **			0.59
**Surplus embryos**	30 (58.8)	26 (45.1)	
**Risk of OHSS **	19 (37.3)	21(41.2)	
**Endometrial polyp**	2 (3.9)	4 (7.8)	


**Table 3:** Frozen-thawed embryo replacement cycle characteristics


Outcome variable	Progesterone N=51	No progesterone N=51	P value

**Endometrial thickness (mm)**	8.7 ± 1.3	8.9 ± 1.4	0.64
**E2 on hCG day (pg/ml)**	208.4 ± 60.2 median: 200	196.9 ± 85.3 median: 170	0.11
**Progesterone on hCG day (ng/ml)**	0.77 ± 0.09	0.80 ± 0.07	0.08
**LH on hCG day (IU/L)**	4.9 ± 1.9	4.6 ± 1.7	0.39
** No. of days until hCG**	14.3 ± 1.8	13.7 ± 1.5	0.07
**No. of embryos transferred**	1.7 ± 0.5 median: 2	1.9 ± 0.5 median: 2	0.07
**Transfers with good quality embryos (%)**	54.9	60.8	0.54


**Table 4:** Pregnancy outcomes


Outcome variable	Progesterone N=51	No progesterone N=51	P value

**Chemical pregnancy rate‚ n (%)**	18 (35.3)	16 (31.4)	0.83
**Clinical pregnancy rate‚ n (%)**	17 (33.3)	14 (27.5)	0.66
**Implantation rate (%)**	16.6	15.3	0.93
**Clinical abortion rate‚ n (%)**	2 (11.8)	2 (14.3)	0.83


## Discussion

The granulosa cells of the developing follicle generate estradiol in response to gonadotropin stimulation in natural cycles. The endometrium acquires receptivity to embryo implantation by responding to progesterone action on an appropriately primed endometrium. Estrogenic stimulation would result in endometrial proliferation and the induction of progesterone receptors. The endometrium undertakes profound conformational and biochemical changes, from proliferative to secretory, with a concomitant induction of endometrial receptivity and opening of the window of implantation in response to progesterone (20). During the implantation window, the endometrium which is unexpectedly unreceptive towards embryo implantation acquires a functional condition useful to blastocyst reception (21).

The transfer of frozen-thawed embryos has important implications for the management of women undergoing ovarian hyperstimulation for IVF (2). Frozen embryo transfer is reported to be successful during the natural cycle after spontaneous ovulation according to the literature (22). In a study by Morozov et al. a higher pregnancy rate was observed in recipients who underwent natural cryothaw cycles than in hormone replacement treatment cycles. In their study the level of estradiol was greater in the substitution cycles when compared with the natural cycle. Regarding those results, we have supported the theory that the window of uterine receptivity closes earlier at a higher endogenous estrogen level and limits the time for the transferred embryos to implant successfully (9). According to their results,hormone replacement treatment versus the natural cycle for cryothaw embryo transfer was associated with decreased pregnancy rates. In the current study, we have evaluated the outcome of hCG-induced natural cryothawed embryo transfer cycles that were supported during the luteal phase with IM progesterone. We compared this with the outcome of hCG-induced natural cycles in the absence of luteal phase support.

Our hypothesis was that progesterone support has a beneficial effect on pregnancy rate after frozen embryo transfer in natural cycles, but the results did not support our hypothesis. In our study, hCG was used for final oocyte maturation. It was suggested that hCG administered for the final oocyte maturation in stimulated IVF cycles would cause a luteal phase defect by suppressing LH production through a short-loop feedback mechanism (23) although the use of hCG did not down-regulate LH secretion in the luteal phase of regular and unstimulated cycles in women with normal ovulation (24). Additionally, in our study none of the patients developed premature luteinization. Premature LH surge is defined as an LH level of ≥10 IU/L and a progesterone level of ≥1.0 ng/ml on the day of hCG administration (25). An elevated progesterone level advances the endometrium‚ therefore the replacement of day 3 embryos occur in an asynchronous endometrium with subsequent failure of establishing an embryo-endometrium cross-dialog, resulting in implantation failure (26).

Bourgain et al. have reported that progesterone induces a secretory transformation of the endometrium in the luteal phase (27) and by inducing this change after sufficient estrogen priming, progesterone improves endometrial receptivity (28). Progesterone not only supports endometrial development but also maintains embryo survival by shifting the immune system toward the production of non-inflammatory Th2 cytokines (29, 30). In addition, by inducing nitric oxide synthesis in the deciduas‚ they intensify local vasodilatation and uterine repose (31). A study by Orvieto et al. has shown that, in artificial cryothawed embryo transfer cycles, a highdose progesterone supplementation in the luteal phase resulted in a higher clinical pregnancy rate (32).

In contrast to our study, Bjuresten et al. have reported that progesterone supplementation improved the live birth rate after embryo transfer in natural cycles (15). In their study, women received vaginal progesterone at a dose of 400 mg twice a day from the day of the embryo transfer. They attributed the increase in live birth rate to the effects of vaginal progesterone. Vaginal progesterone results in adequate endometrial development, in spite of low serum progesterone levels.

Our study was in agreement with a study by Kyrou et al. that reported luteal phase support did not affect ongoing pregnancy rates in natural hCG-induced frozenthawed embryo transfer cycles (33). A possible reason for our finding was that the women in the present study had a normal ovulatory function; those with ovulatory dysfunction were excluded from the study. Luteal phase defect in stimulated IVF cycles is due to supra physiological levels of steroids which directly inhibit the LH release via negative feedback actions at the hypothalamic- pituitary axis level (34). However it seems that LPD is not a main etiologic factor for implantation failure in natural frozen thawed embryo transfer cycles.

## Conclusion

There emerged no significant differences between the two groups in our study with regards to the implantation or clinical pregnancy rates‚ but there was a trend toward an increased clinical pregnancy rate with luteal supplementation. Thus, further studies are needed to confirm our findings.
